# Integration of measurable residual disease by WT1 gene expression and flow cytometry identifies pediatric patients with high risk of relapse in acute myeloid leukemia

**DOI:** 10.3389/fonc.2024.1340909

**Published:** 2024-04-24

**Authors:** Sonia Ahmed, Mariam Elsherif, Dina Yassin, Nahla Elsharkawy, Ayman S. Mohamed, Nouran Yasser, Amr Elnashar, Hanafy Hafez, Edward A. Kolb, Alaa Elhaddad

**Affiliations:** ^1^ Department of Pediatric Oncology, National Cancer Institute, Cairo University, Cairo, Egypt; ^2^ Department of Pediatric Oncology, Children’s Cancer Hospital Egypt (CCHE-57357), Cairo, Egypt; ^3^ Department of Clinical Pathology, Children’s Cancer Hospital Egypt (CCHE-57357), Cairo, Egypt; ^4^ Department of Clinical Pathology, National Cancer Institute, Cairo University, Cairo, Egypt; ^5^ Department of Research and Biostatistics, Children’s Cancer Hospital (CCHE-57357), Cairo, Egypt; ^6^ Department of Pediatric Hematology and Oncology, Nemours Center for Cancer and Blood Disorders, Wilmington, DE, United States; ^7^ Leukemia and Lymphoma Society, Rye Brook, NY, United States

**Keywords:** *WT1* gene overexpression, measurable residual disease, pediatric AML, outcome, flow cytometry

## Abstract

**Background:**

Molecular testing plays a pivotal role in monitoring measurable residual disease (MRD) in acute myeloid leukemia (AML), aiding in the refinement of risk stratification and treatment guidance. Wilms tumor gene 1 (*WT1*) is frequently upregulated in pediatric AML and serves as a potential molecular marker for MRD. This study aimed to evaluate *WT1* predictive value as an MRD marker and its impact on disease prognosis.

**Methods:**

Quantification of *WT1* expression levels was analyzed using the standardized European Leukemia Network real-time quantitative polymerase chain reaction assay (qRT-PCR) among a cohort of 146 pediatric AML patients. Post-induction I and intensification I, MRD response by *WT1* was assessed. Patients achieving a ≥2 log reduction in *WT1*MRD were categorized as good responders, while those failing to reach this threshold were classified as poor responders.

**Results:**

At diagnosis, *WT1* overexpression was observed in 112 out of 146 (76.7%) patients. Significantly high levels were found in patients with M4- FAB subtype (p=0.018) and core binding fusion transcript (CBF) (*RUNX1::RUNX1T1*, p=0.018, *CBFB::MYH11*, p=0.016). Following induction treatment, good responders exhibited a reduced risk of relapse (2-year cumulative incidence of relapse [CIR] 7.9% *vs* 33.2%, p=0.008). Conversely, poor responders’ post-intensification I showed significantly lower overall survival (OS) (51% *vs* 93.2%, p<0.001), event-free survival (EFS) (33.3% *vs* 82.6%, p<0.001), and higher CIR (66.6% *vs* 10.6%, p<0.001) at 24 months compared to good responders. Even after adjusting for potential confounders, it remained an independent adverse prognostic factor for OS (p=0.04) and EFS (p=0.008). High concordance rates between *WT1*-based MRD response and molecular MRD were observed in CBF patients. Furthermore, failure to achieve either a 3-log reduction by RT-PCR or a 2-log reduction by *WT1* indicated a high risk of relapse. Combining MFC-based and *WT1*-based MRD results among the intermediate-risk group identified patients with unfavorable prognosis (positive predictive value [PPV] 100%, negative predictive value [NPV] 85%, and accuracy 87.5%).

**Conclusion:**

*WT1*MRD response post-intensification I serves as an independent prognostic factor for survival in pediatric AML. Integration of *WT1* and MFC-based MRD results enhances the reliability of MRD-based prognostic stratification, particularly in patients lacking specific leukemic markers, thereby influencing treatment strategies.

## 1 Introduction

The survival rate of pediatric patients with acute myeloid leukemia (AML) approached 70%, with approximately 30% experiencing relapse, which is the main cause of treatment failure (1). Measurable residual disease (MRD) is an important biomarker in AML patients used for prognosis and response assessments (2).

The prognostic value of MRD detected by real-time quantitative polymerase chain reaction (RT-qPCR) or multiparametric flow cytometry (MFC) is well established, and relapse occurs more likely in patients with detectable MRD (3). Previously published data suggested that patients with one positive and one negative MRD results performed by two different techniques have higher relapse risk than patients with two negative MRD results, but lower relapse risk than patients with two positive MRD (2, 4, 5).

Specific genetic aberrations, such as *RUNX1::RUNX1T1*, *CBFB::MYH11*, and nucleophosmin 1 *(NPM1)* mutations, constitute markers of MRD but about 40% of children with AML harbor leukemia-specific targets, which makes these genetic targets clinically applicable in only minor fraction of children (6, 7). Therefore, it was crucial to identify other molecular targets applicable to the majority of patients.

Wilms tumor gene 1 (*WT1*) is overexpressed in approximately 80% of children with AML. Previous cohort studies in children and adults evaluated *WT1* gene overexpression at diagnosis, during treatment, and pre-, and post-hematopoietic stem cell transplantation (HSCT) to provide a target for novel immunotherapeutic approaches and to advocate it as a universal marker for MRD assessment.

The European Leukemia Network researchers (ELN) validated a quantitative *WT1* assay and established reference ranges for *WT1* expression in peripheral blood (PB) and bone marrow (BM) by analyzing a large number of control samples. This study showed that (≥2 log) reduction in *WT1* transcript after induction predicted reduced relapse risk, also failure to reduce *WT1* transcripts below this threshold limits after intensification predicted increased risk of relapse (p=.004) (8). For pediatric AML, Lapillonne et al. observed that *WT1* level higher than 50 × 10^4^ after induction was an independent prognostic risk factor of relapse (p=.002) and death (p=.02) (9).

A recent consensus from the ELN MRD working group recommends *WT1* as an important tool in monitoring MRD and stratifying patients with AML especially those who lack a more sensitive marker and thus could influence treatment strategy (2).

The objective of this study was to assess the prevalence of *WT1* gene overexpression in pediatric patients with AML treated at Children’s Cancer Hospital Egypt (CCHE-57357) on AML protocol adopted from the Children Oncology Group (COG) (NCT01371981) (10) and to evaluate the prognostic significance of *WT1* as an MRD marker on survival and disease outcome.

## 2 Patients and methods

This study comprised 163 pediatric patients with *De novo* AML diagnoses who received therapy at CCHE-57357 between January 2019 and May 2020. A minimum follow-up period of one year from the completion of treatment was ensured for all participants. The study was approved by the Institutional Review Board (IRB), and informed consent was secured prior to the start of therapy.

Patients with conditions including acute promyelocytic leukemia (APL), Down syndrome myeloid neoplasm, myelodysplastic syndrome (MDS), therapy-related AML (t-AML), and myeloid sarcoma were excluded from the study. Data related to the patients were gathered from their electronic medical records and initial disease assessments included morphology, immune-phenotyping, cytogenetics, and molecular studies. For disease evaluation bone marrow aspirate (BMA) was done after each cycle and MRD was monitored by MFC based on the ELN proposed consensus using 8-10 colors monoclonal antibody (mAbs) panels. We adopted two methodologies: the Leukemia Associated Immunophenotype (LAIP) approach and the Different from Normal (DFN) approach. MRD is detected based on LAIP’s present on any population and any deviation seen from normal patterns (7, 11). For patients with core binding fusion transcript (CBF), molecular MRD assessment by PCR was done post-intensification I.

Patients were enrolled on the CCHE 57357 AML protocol, which was adapted from the modified COG protocol (NCT01371981) (10) and based on their cytogenetics and molecular abnormalities, patients were categorized into Low risk (LR) groups: Patients with favorable cytogenetics, including. CBF (t(8;21) (q22;q22.1); *RUNX1::RUNX1T1*, inv (16) (p13.1;q22) or t (16;16) (p13.1;q22); *CBFB::MYH11*), Nucleophosmin 1 (*NPM1*), and *CEBPA* abnormalities. Intermediate risk (IR): Patients without either favorable or unfavorable criteria. High risk (HR): Patients with unfavorable cytogenetics, such as monosomy 7, monosomy 5, complex karyotype (more than three chromosomal aberrations), and FLT3 internal tandem duplications (*FLT3/ITD*) with a high allelic ratio (> 0.4, *FLT3/ITD* positive). Additionally, IR patients were further classified into HR and LR categories based on MRD response measured by MFC after the first induction. LR was defined as MRD <0.1% (10).

All patients received 4 cycles of chemotherapy and only high-risk were offered allogeneic hematopoietic stem cell transplantation (Allo-HSCT) in complete remission (CR) if a matched sibling donor is available. The chemotherapy regimen details and dosages are given in “**Supplementary Table 1**”.

### 2.1 Definitions

Complete remission (CR) was defined as bone marrow (BM) blasts <5%. Refractory disease was defined as the persistence of blasts ≥ 5% at the end of induction II. Relapse was defined as the reappearance of leukemic blasts in the peripheral blood or >5% blasts in the bone marrow after achieving CR. Disease-Related Mortality (DRM): Death within the first 14 days of induction I (early deaths) or with evidence of disease in the last evaluation. Treatment-Related Mortality (TRM): Death beyond the first 14 days of induction I or death from any cause other than disease in subsequent cycles (without evidence of morphological disease).

### 2.2 Real-time quantitative PCR for *WT1* gene overexpression

Since *WT1* is normally expressed in hematopoietic cells it is critical to establish the level of expression seen in normal control samples so that a threshold can be defined that distinguishes between residual leukemia and background amplification. We used the ELN assay incorporated in the ipsogen^®^ *WT1* ProfileQuant^®^ Kit (CE-approved kit, Ref 676923, QIAGEN GmbH). The median *WT1* expression level was 19.8 copies/10^4^ *ABL* copies (with a range of 0–213 copies) in normal bone marrow samples. The upper limit of normal as 250 normalized *WT1* copies (NCN) for bone marrow samples was established. This threshold was selected for its optimal sensitivity and specificity ensuring accurate discrimination between normal and abnormal *WT1* expression levels (8). Pure RNA was extracted using a total RNA Purification Kit following the manufacturer protocol (QIAamp RNA Blood Mini Kit). Thermo Scientific NanoDrop 2000 was used to quantify and assess the purity of all RNA samples to be sure that the concentrations were pure enough to conduct RT-PCR. RNA input for all samples was adjusted (1 μg) and cDNA was synthesized using a Reverse transcription kit (Thermo Fisher Scientific, High-Capacity cDNA Reverse Transcription Kit). Quantitative mRNA expression study of the *WT1* gene was carried out relative to the expression of a housekeeping gene *ABL* by QuantStudio™ 5 RT-PCR System (Applied Biosystems™) using Thermo Fisher Scientific, TaqMan™ Universal PCR Master Mix. cDNA input for all samples was adjusted (100 ng/μl), the threshold value was adjusted to 0.1, and the expression was measured using absolute quantification (standard curve). Bone marrow samples with *WT1* expression > 250 × 10^4^ *ABL* copies at diagnosis were considered to have overexpression and designated as *WT1* ^+ve^ while the remaining samples were referred to as *WT1* ^-ve^. The Magnitude of log reduction in *WT1* transcripts level after induction I and Intensification I was measured by RT-qPCR using ELN (8). Patients with *WT1* MRD ≥2 log reduction were considered good responders (*WT1* MRD^-ve^) and those with *WT1* MRD <2 log were poor responders (*WT1* MRD^+ve^). **Supplementary Table 2** displays the primers and probes used for ABL and *WT1*.

## 3 Statistical methodology

The patients’ characteristics were compared using the chi-square test and Mann-Whitney test with Bonferroni multiple hypothesis *P*-value adjustment. Overall, event-free, and relapse-free survival probabilities (OS, EFS, RFS) were calculated using Kaplan-Meier analysis, and comparisons between patients were performed using the log-rank test. OS was defined as the interval from the date of diagnosis until the date of death or last contact date. EFS was delineated as the interval from the date of diagnosis until the occurrence of an event, which could be relapse, refractory, or death. RFS was defined as the period from the date of achieving complete remission to the date of relapse or death. The impact of various risk factors on both death and events was assessed through univariate Cox regression. A deliberate selection process was employed to choose variables for reevaluation using a multivariate Cox regression model. The gray test was utilized to estimate and compare the cumulative incidence of relapse (CIR).

## 4 Results

### 4.1 Prevalence of *WT1* gene overexpression and its correlation with initial patient characteristics.

**Figure 1:**
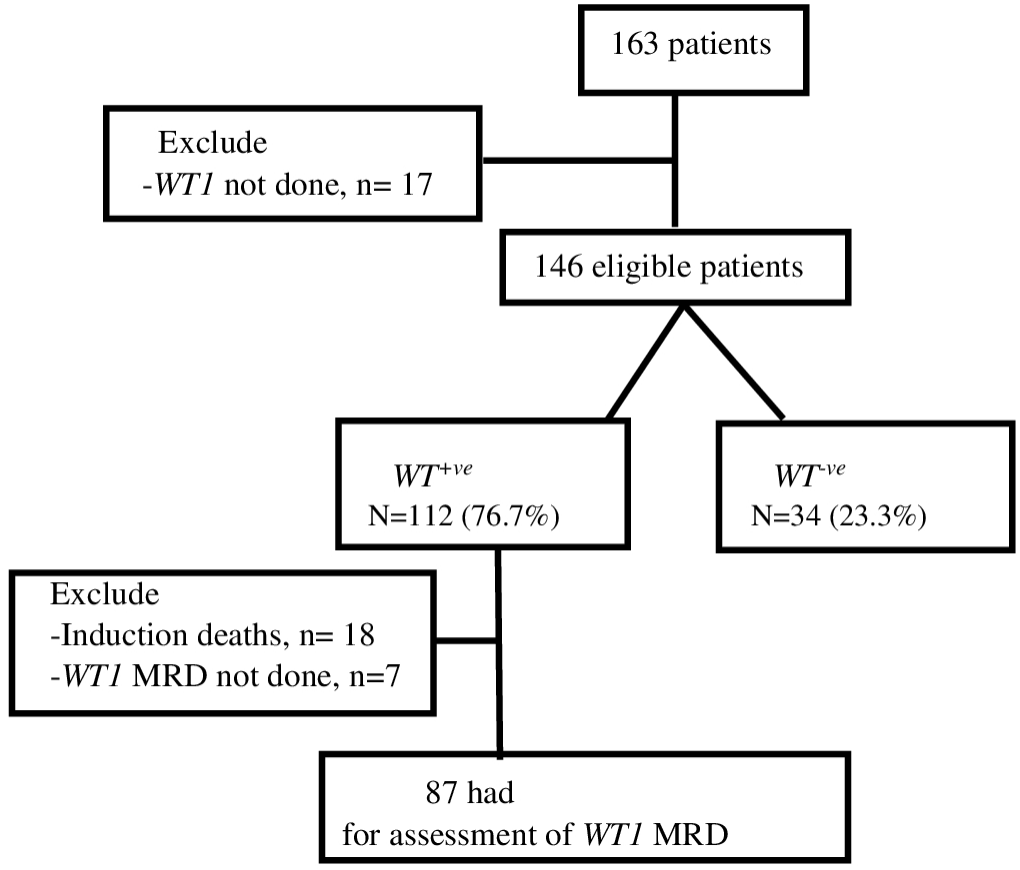
Prevalence of *WT1* gene overexpression among pediatric patients with AML.

Out of the 146 eligible patients, 112 (76.7%) exhibited *WT1* overexpression (refer to **Figure 1**). The median age for the entire cohort was 8.8 years (range, 0.3–17.9 years). Patients with *WT1* overexpression at diagnosis had higher median age (range, 0.3–17.9 years) (55% of *WT1* ^+ve >^8.8 years, p=0.014). Levels were significantly higher in the M4 FAB AML subtype (n= 32/35 (91.4%) p=0.018), and patients with CBF (t (8:21) n= 32/35 (91.4%), p=0.018; inv 16, n= 17/17 (100%) p=0.016). The rate of *KMT2A* gene rearrangements was lower in patients with *WT1* overexpression (4/14 (28.5%), p<0.001). No significant association was found between unfavorable cytogenetics (FLT3/ITD, complex karyotype, monosomy 7 and monosomy 5), Trisomy 8 or Trisomy 21, and *WT1* overexpression. According to the COG initial risk classification, High *WT1* levels were more prevalent in the LR compared to IR and HR groups (52% of *WT1* ^+ve^ were LR *vs*. 36% were IR and 15% were HR, p<0.001) (**Table 1**).

**Table 1:** Distribution of *WT1* gene overexpression depending on initial patient’s characteristics.

	Total No.	*WT1* expression		P-value
	Whole cohort	Yes	No	
	No=146 N (%)	No= 112 N (row %)	No= 34 N (row %)	
Age				0.014*
Median (IQR)**	8.8	9.3	5.2	
Range	0.3-17.8	0.3 – 17.8	0.7- 16.7	
Total Leukocytic count group				0.293
<50	120 (82.2%)	90 (75%)	30 (25%)	
>50	26 (17.8%)	22 (84.6%)	4 (15%)	
FAB				
M0	10 (6.8%)	3 (30%)	7(70%)	<0.001*
M1	24 (16.4%)	20 (83.3%)	4 (16.7%)	0.401
M2	45 (30.8%)	39 (86.7%)	6 (13.3%)	0.05
M4	35 (24%)	32 (91.4%)	3 (8.6%)	0.018*
M5/5a	16 (11%)	8 (50%)	8 (50%)	0.007*
M6	0 (0.0%)	–	–	NA
M7	16 (11%)	10 (62.5%)	6 (37.5%)	0.154
Cytogenetics and molecular				
Favorable				
-t(8:21)	35 (24%)	32 (91.4%)	3 (8.6%)	0.018*
-Inversion 16	17 (11.6%)	17 (100%)	0 (0.0%)	0.016*
-NPM1/CEBPA				0.775
NPM1	6 (4.1%)	5 (83.3%)	1 (16.6%)	
CEBPA	5 (3.4%)	4 (80%)	1 (20%)	
Intermediate				
-Normal karyotype	32 (22%)	20 (62.5%)	12 (37.5%)	0.157
-*KMT2A-r*	14 (9.5%)	4 (28.5%)	10 (71.4%)	<0.001*
-Trisomy 8	10 (7%)	8 (80%)	2 (20%)	0.677
-Trisomy 21	6 (4.1%)	6 (100%)	0 (0.0%)	0.168
-t(1:22)	4 (2.7%)	4 (100%)	0 (0.0%)	0.264
Unfavorable				
-Complex karyotype	6 (4.1%)	5 (83.3%)	1 (16.6%)	0.775
-Monosomy 7	5 (3.4%)	5 (100%)	0 (0.0%)	0.135
-Monosomy 5	3 (2%)	1 (33.3%)	2 (66.7%)	0.072
-t (6:9)	1 (0.68%)	1 (100%)	0 (0.0%)	0.580
-FLT3/ITD	4 (2.7%)	4(100%)	0 (0.0%)	0.264
Initial risk stratification				<0.001*
-Low risk	63 (43.2%)	58 (92.1%)	5 (7.8%)	
-Intermediate	66 (45.2%)	40 (60.6%)	26 (39.3%)	
-High risk	17 (11.6%)	14 (82.3%)	3 (17.6%)	

*Significant; P-value < 0.05.

**IQR: interquartile range.

NPM1, Nucleophosmin 1; CEBPA, CCAAT enhancer-binding protein alpha.

### 4.2 Survival analysis

**Figure 2:**
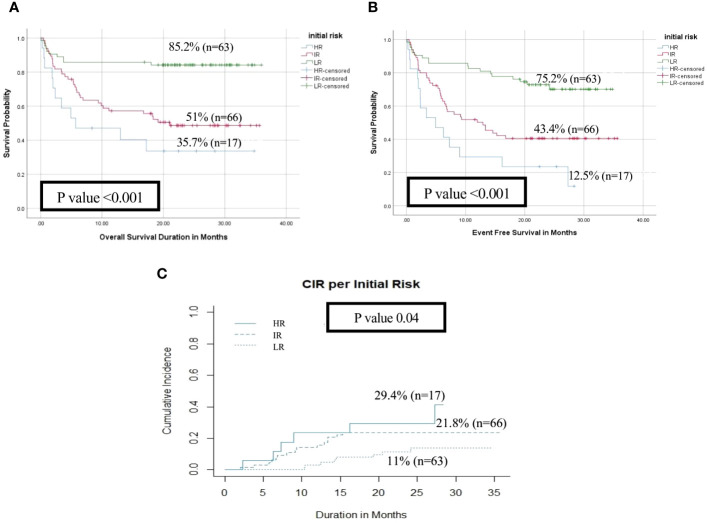
Impact of initial risk stratification on the disease outcome of pediatric patients with AML (HR n=17 vs IR n=66 vs LR n=63). **(A)** Overall survival, **(B)** Event-free survival, **(C)** Cumulative incidence of relapse (CIR).

**Figure 3:**
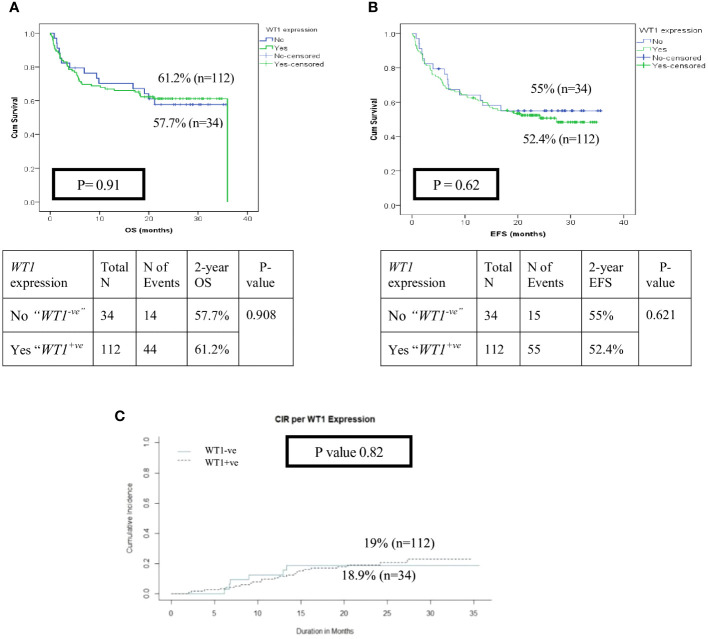
Impact of *WT1* overexpression (*WT1^+ ve^* vs *WT1^-ve^*) at diagnosis on the disease outcome of pediatric patients with AML **(A)** Overall survival of patients with *WT1^+ve^* vs *WT1^-ve^*, **(B)** Event-free survival of patients with *WT1^+ve^* vs *WT1^-ve^*, **(C)** Cumulative incidence of relapse (CIR).

With a median follow-up of 21 months, the overall survival (OS) and the event-free survival (EFS) of the entire cohort at 2 years were 64.5% and 55.3% respectively. HR patients had significantly lower OS, EFS, and higher cumulative incidence of relapse at 2- year (OS; 35.7%, EFS; 12.5% and CIR; 29.4%) than patients in the IR (OS; 51%, EFS; 43.4%, CIR; 21.8%) and LR groups (OS; 85.2%, EFS; 75.2%, CIR; 11%, p<0.001) (**Figures 2A–C**). There was no significant impact for *WT1* overexpression at diagnosis on survival and relapse at 2 years when compared to patients with low *WT1* expression level (OS; *WT^+ve^*; 61.2% *vs WT^-ve^*; 57.7%, p= 0.9*)*, (EFS; *WT^+ve^*; 52.4% *vs*. *WT^-ve^*; 55%, p=0.621) (CIR; *WT^+ve^*; 19% *vs*. *WT^-ve^*; 18.9%, p=0.82) (**Figures 3A–C**).

### 4.3 Outcome-based on MRD response by *WT1*

**Figure 4:**
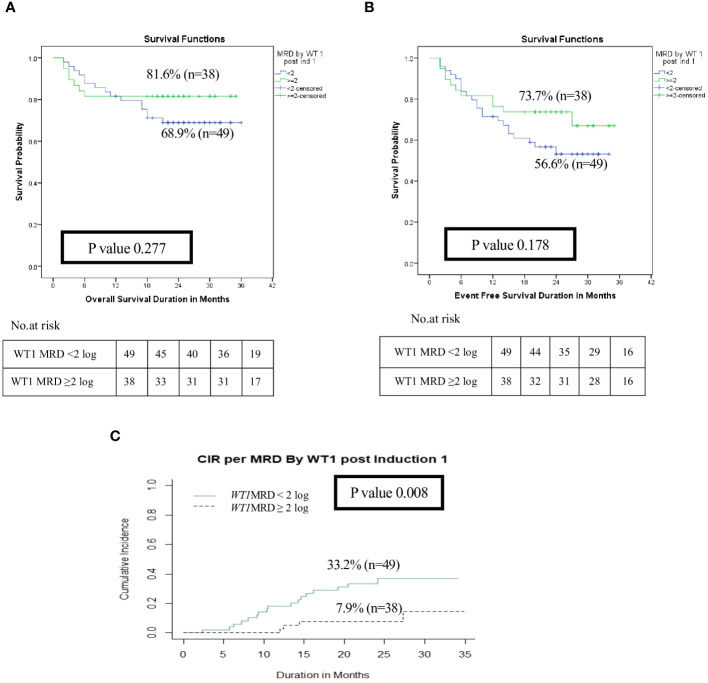
Impact of MRD response by *WT1* on the disease outcome of pediatric patients with AML pediatric patients with AML post Induction I (*WT1* MRD response <2 log reduction vs ≥ 2log reduction). **(A)** Overall survival, **(B)** Event-free survival, **(C)** Cumulative incidence of relapse (CIR).

**Figure 5:**
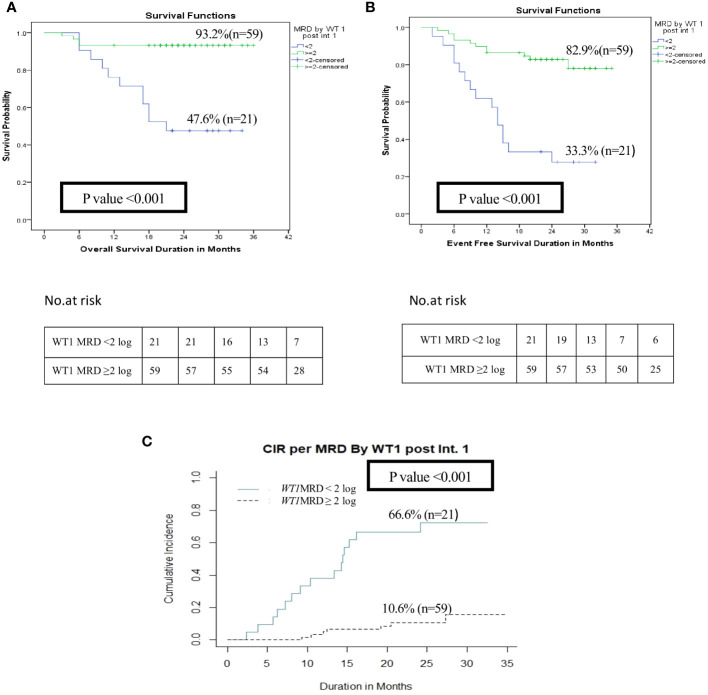
Impact of MRD response by *WT1* on the disease outcome of pediatric patients with AML pediatric patients with AML post-intensification I (*WT1* MRD response <2 log reduction vs ≥2 log reduction). **(A)** Overall survival, **(B)** Event-free survival, **(C)** Cumulative incidence of relapse (CIR).

After exclusion of induction deaths (n=18) and patients without available MRD data (n=7), patients with *WT1* MRD ≥2 log reduction (*WT1* MRD^-ve^) after induction I showed a significant decrease in the risk of relapse when compared to those with *WT1* MRD <2 log reduction (*WT1* MRD^+ve^) (2-year CIR was 7.9% for *WT1* MRD^-ve^ *vs*. 33.2% for *WT1* MRD^+ve^, p=0.008), however there was no difference in OS and EFS among each group (**Figures 4A–C**, **Supplementary Table 3**). Additionally, poor responders by *WT1* MRD (<2 log reduction) after intensification I had significantly lower 2 years OS (47.6% *vs*. 93.2%, p<0.001) and EFS (33.3% *vs*. 82.6%, p<0.001), and significantly higher CIR at 2 years (66.6% *vs*. 10.6%, p<0.001) (**Figures 5A–C**, **Supplementary Table 3**) .

### 4.4 Univariate and multivariate Cox regression analysis

*WT1* MRD post intensification I, FAB M7, favorable cytogenetics, unfavorable cytogenetics, and risk stratification post induction 1 were identified as significant predictors for survival rates in univariate analysis. Regression multivariate analysis demonstrated that post intensification I, poor response by *WT1* MRD (<2 log reduction) was an independent poor prognostic marker on survival (OS Hazards ratio (HR), 3; Confidence Interval (CI), 0.9 –10.6; p=0.04 and EFS HR, 3.115; CI range, 1.35—7.19, p= 0.008). Also risk stratification post induction I based on MRD response by MFC had a significant impact on survival (OS HR, 18.05; CI range, 2.22-146.6; p=0.007) and EFS (EFS HR, 2.83; CI range, 1.1.7—6.82, p=0.02) (**Supplementary Tables 4A, B**). Regarding the relapse free survival (RFS), in univariate and multivariate analysis, *WT1*MRD response post intensification I and initial risk group remained as a significant prognostic factor (p=0.01, p=0.02 respectively) (**Table 2**).

**Table 2:** Cox regression for univariate and multivariate analyses of MRD by *WT1* post intensification 1, MFC MRD post intensification 1, and Initial risk.

Relapse Free Survival	Univariate				Multivariate			
	Hazards ratio	95% Confidence interval		P-value	Hazards ratio	95% Confidence interval		P-value
-*WT1* MRD post int1 (<2 vs >=2)	8.623	1.892	19.421	<0.001*	6.761	2.533	18.046	0.01*
-MFC MRD post int 1 (<0.1% vs >=0.1%)	0.974	1.174	4.972	0.278				
Initial risk stratification -LR -IR -HR	1(–) 3.8 7.23	1(–) 2.11 3.45	13.81 15.18** **	<0.001*	1(–) 1.505 4.192	1(–) 0.543 1.192	4.177 14.743	0.025*

*Significant; P-value < 0.05; Int 1, intensification I.

### 4.5 Impact of *WT1* MRD response on relapse among LR and IR patients

**Figure 6:**
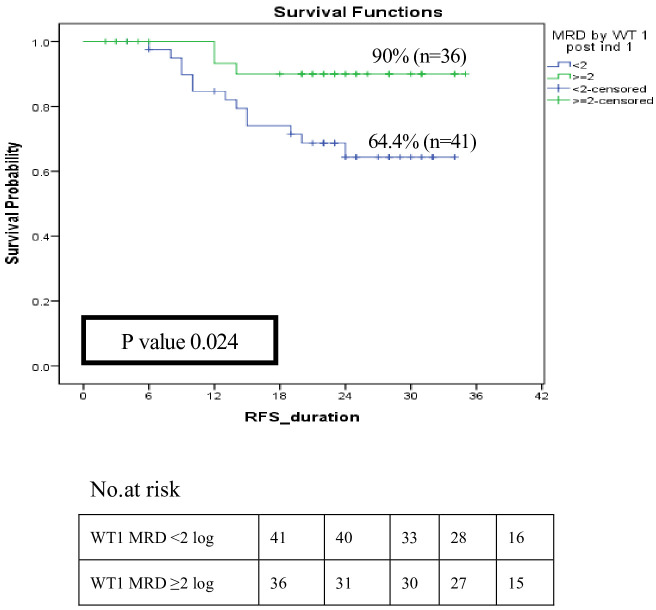
Impact of MRD response by *WT1* on relapse-free survival among low and intermediate risk groups post induction I (*WT1* MRD response < 2 log reduction vs *WT1* MRD ≥ 2log reduction).

The role of *WT1* as an MRD marker and its influence on relapse among LR and IR patients (n=98) was evaluated, 77 patients had available MRD results by both MFC and *WT1*. The current results revealed that good responders (*WT1*MRD^-ve^) post-induction I had a significantly better RFS compared to poor responders (*WT1*MRD^+ve^) at 2 years (90% *vs*. 64.4%, p=0.024) (**Figure 6**). Moreover, within patients with MRD by MFC post-induction I >0.1, *WT1*MRD^-ve^ patients were associated with a significantly lower incidence of relapse compared to those with *WT1*MRD^+ve^ (8.5% *vs*. 30%, p=0.021) (**Supplementary Table 5**).

**Figure 7:**
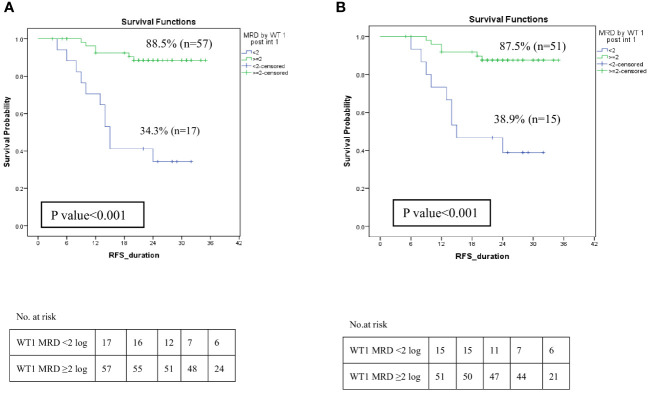
Impact of MRD response by *WT1* on relapse-free survival among LR and IR groups post Intensification I. **(A)** Relapse-free survival of patients with *WT1*MRD response <2 log reduction vs *WT1*MRD ≥2 log reduction, **(B)** Relapse-free survival among patients with negative MRD by MFC (<0.1%) post intensification I (*WT1*MRD response <2 log reduction vs *WT1*MRD ≥2 log reduction).

Further analysis to assess the impact of MRD response by *WT1* post intensification I was investigated. *WT1*MRD^+ve^ patients demonstrated a significant decrease in RFS at 2-year 34.3% compared to 88.5% for *WT1*MRD^-ve^ patients (p<0.001) (**Figure 7A**). Moreover, failure to achieve a 2-log reduction by *WT1* MRD post-intensification I identified patients at a higher risk of relapse, despite having negative MRD by MFC, with rates of 60% compared to 9.8% (p<0.001) (**Supplementary Table 6**). Furthermore, these patients had significantly lower RFS (38.9% compared to 87.5%, p<0.001) (**Figure 7B**).

Descriptive analysis for patients with CBF and *WT1* was done (n=40). Post intensification, 34 patients achieved MRD negativity by both *WT1* and PCR and none of them relapsed. While among the remaining 6 patients with MRD by *WT1* (<2 log reduction) and PCR (<3 log reduction), 50% relapsed. Despite the small number of patients precluded detailed statistical analysis, the high concordance rate confirmed that MRD evaluation by molecular testing at this time point is crucial as it predicts relapse and failure to achieve either 3 log by PCR or 2 log by *WT1* carries a high incidence of relapse (**Supplementary Table 7**).

In terms of intermediate-risk patients, *WT1*MRD showed greater sensitivity compared to MFC (77.8% *vs*. 22.2%). Notably, patients with *WT1*MRD positivity had the highest risk of relapse, with a Positive Predictive Value (PPV) of 63.6% and a Negative Predictive Value (NPV) of 87.5%. Contrarily, patients with undetectable MRD by MFC experienced a reduced risk of relapse, with a NPV of 72% and a specificity of 90%. The combination of both MRD assessments enabled patients to capitalize on the high sensitivity of *WT1*MRD and the high specificity of MFC MRD, effectively stratifying patients into three distinct risk group: negative MRD by both *WT1* and MFC, *WT1* MRD^-ve^ but MFC MRD^+ve^, and positive MRD by both *WT1* and MFC (**Table 3**).

**Table 3:** Comparing between MRD by MFC and *WT1* MRD among IR group post intensification I.

		Non-relapse	Relapse	Sensitivity	Specificity	PPV*	NPV**	Accuracy
		N (%)	N (%)					
MRD MFC***	< 0.1% (-ve)	18 (90.0%)	7 (77.8%)	22.20%	90.00%	50.00%	72.0%	68.97%
	≥ 0.1% (+ve)	2 (10.0%)	2 (22.2%)					
WT 1 MRD****	Good responder (≥2 log) (-ve)	14 (77.8%)	2 (22.2%)	77.80%	77.80%	63.64%	87.50%	77.78%
	Poor responder (<2 log) (+ve)	4 (22.2%)	7 (77.8%)					
Combination	Good	12 (100.0%)	2 (50.0%)	50.0%	100.0%	100.0%	85.71%	87.5%
	Poor	0 (0.0%)	2 (50.0%)					

*PPV, Positive predictive value; MRD MFC ≥ 0.1%, WT1MRD < 2 log reduction and relapsed.

**NPV, Negative predictive value; MRD MFC < 0.1%, WT1MRD ≥2 log reduction and didn’t relapse.

***MRD MFC, Measurable residual disease by multiparametric flow cytometry.

**** WT1MRD, Measurable residual disease by WT1 gene expression.

The MRD response assessed by *WT1* can serve as a valuable tool to pinpoint patients at a high risk of relapse, particularly among pediatric patients lacking identified markers for monitoring. Additionally, it effectively identifies patients within the intermediate-risk (IR) group with a reduced incidence of relapse, thereby influencing treatment strategies. However, to validate these findings and ensure their reliability in clinical decision-making, a larger cohort of patients should be examined.

## 5 Discussion

Measurable residual disease (MRD) by MFC is a strong and independent prognostic marker of relapse in pediatric AML, yet they are still not sensitive enough, since relapse still occurs in a minority of MRD-negative patients and the opposite is true, not all patients with MRD-positive will relapse (12). Therefore, searching for more sensitive molecular markers is of great importance and molecular evidence of residual leukemia cells can predict relapse several months before clinical emergence (13, 14).

The incidence of *WT1* gene overexpression in pediatric AML, as reported in previous studies, ranges from 76% to 83% (9, 15–17), which aligns with the current results. However, Juul-Dal et al. showed a much lower incidence of *WT1* overexpression (45%) (1). Previous pediatric studies showed that *WT1* gene overexpression was significantly associated with M4 FAB subtype, this goes in agreement with our results (91.4% of M4 cases had high *WT1* levels, p=0.018) (9, 18, 19). A high incidence of *WT1* overexpression in CBF AML was reported, ranging from 87% up to 100% (p<0.001), while a strong inverse correlation with the presence of *KMT2A* gene rearrangement (p<0.001) suggesting downregulation of *WT1* activating pathway in this leukemia subset, as demonstrated in the present study (1, 9, 20).

The ELN in 2021 recommended *WT1* gene expression as a marker for MRD assessment especially in patients without identified markers to follow (2, 7). The current results suggested that failure to achieve 2 log reduction by *WT1* MRD post induction I predict relapse (2-year CIR was 33.2% *vs* 7.9%, p=0.008), but this wasn’t reflected on survival analysis which may be explained by treatment-related mortalities during induction. Previous data published by Lapillonne et al. demonstrated that *WT1* levels higher than 50 × 10^4^ *ABL* copies after induction was an independent prognostic risk factor of relapse (p = 0.002) and death (p = 0.02*)* in pediatric AML (9). While Shimada et al. reported that high *WT1* expression after 1^st^ induction chemotherapy would be associated with poor outcomes in pediatric AML patients, (5-year OS for *WT1* ^+ve^ was 54.5% *vs* 79.4% for *WT1* ^-ve^ patients, p=0.036), but multivariate analyses did not confirm it as an independent poor prognostic factor on the outcome of AML patients (OS, p=0.87 and EFS, p=0.92) (21).

In this study, poor responders (*WT1* MRD < 2log reduction) post intensification I exhibited notably reduced OS and EFS compared to those with *WT1*MRD^-ve^ mostly attributed to a higher cumulative incidence of relapse, which retained its significance as a predictive factor for inferior survival even after accounting for other variables, including initial risk stratification.

These findings align with Cilloni et al. who reported that patients who failed to achieve 2 log reduction by *WT1* MRD after intensification therapy were associated with a significant increase in relapse risk (67% *vs* 42% at 5 years, p= 0.004) (8). Additionally, Nomdedéu et al. analyzed 584 patients under the CETLAM protocol, categorizing them into three groups based on post-intensification *WT1* levels (<10, 10.1 to 100, and >100/10^4^ *ABL*), the OS and LFS were significantly lower among patients with *WT1* >100 copies (OS 30%, LFS 24% and CIR 25%, p<0.001) (22).

Furthermore, Candoni et al. reported the impact of MRD negativity before allo-SCT (post intensification I) on the outcome and found that patients with *WT1* MRD^+ve^ (*WT1* > 250 copies/10^4^ *ABL*) had significantly lower OS (HR: 3.9, p<0.0001), DFS (HR: 3.73, p<0.0001) and higher cumulative incidence of relapse (HR 5.06, p< 0.0001) compared to MRD negative patients (23).

One of the main objectives of our study was to pinpoint a subset of patients, especially within the intermediate-risk (IR) group who are at a higher risk of relapse and poorer outcomes. Additionally, we aimed to investigate the concordance between MRD results by MFC and *WT1*MRD to set the stage for different approaches to upfront therapy for this group. We observed that patients with positive MRD by MFC (≥ 0.1%) after induction I but achieved favorable response by *WT1*MRD had a significantly lower incidence of relapse. Similarly, Marani et al. demonstrated that flow MRD post-induction did not predict DFS. Approximately 40% of flow-MRD^+ve^ patients-maintained CR (p=0.41). In contrast, the response by *WT1* MRD response was predictive (DFS 46% for *WT1*MRD^-ve^ *vs* 0% for *WT1*MRD ^+ve^, p<0.001) (24). After intensification I, failure to reduce *WT1* transcript level identifies a group of patients at significantly increased risk of relapse despite having negative MRD by MFC (RR 60% *vs* 9.8% with p<0.001; (RFS 38.9% *vs* 87.5%, p<0.001).

When comparing *WT1* as an MRD marker with other monitoring techniques such as PCR for fusion gene transcripts *(RUNX1::RUNX1T1, and CBFB::MYH11)*, similar sensitivities were observed in predicting the relapse among CBF pediatric patients with AML and failure to achieve either 3 log by PCR or 2 log by *WT1* post intensification I carry a higher incidence of relapse regardless response by MFC. Similarly, ELN 2021 stated the importance of molecular monitoring of MRD in CBF patients especially at this time point (2).

The current study demonstrates that combined assessment of MRD by *WT1* and MFC ably detected patients at a higher risk of relapse (PPV 100%) and identified patients with low relapse risk (NPV 85.7%) as *WT1*MRD had higher sensitivity while MFC had better specificity among intermediate risk group even with the small number of patients. The same was reported by Guolo et al, where patients with *WT1* MRD positive after consolidation therapy had the highest risk of relapse (negative predictive value (NPV): 79%; positive predictive value (PPV): 62%) whereas patients with undetected MFC MRD had a very low relapse risk (NPV: 92%, PPV: 30%) and combination allowed patients to benefit from both the high PPV of *WT1* MRD and the high NPV of MFC MRD (25).

Carlo Marani et al. concluded that integrating cytogenetic risk at diagnosis with MRD assessment using both flow cytometry and *WT1* allowed for better classification of AML patients: into three prognostic groups: good (no-high risk features “HR” and flow-MRD^-ve^), intermediate (no-HR, flow-MRD^+ve^ and *WT1* ≥2 log) and adverse prognosis (HR or *WT1* < 2 log) with a 3-year DFS of 78.8%, 51.6% and 0%, respectively, p<.001) (24).

In Conclusion, monitoring measurable residual disease by *WT1* expression level either alone (especially in pediatric AML patients without other biological markers) or in combination with other MRD markers may improve the reliability of MRD-based prognostic stratification and thus enable the tailoring of treatment intensity. In addition, suboptimal molecular MRD response (defined as <3 log reduction by qPCR or <2 log reduction by *WT1*) in AML pediatric patients with CBF may influence frontline therapy decisions due to the increased risk of relapse. Therefore, all study groups now apply extensive biological characterization of the AML cells and response based on MRD assessments by both MFC and molecular technique for better risk-group adapted treatment (26).

## Data availability statement

The original contributions presented in the study are included in the article/**Supplementary Material**. Further inquiries can be directed to the corresponding author.

## Ethics statement

The studies involving humans were approved by Children’s Cancer Hospital Egypt Scientific Advisory Committee (SMAC) and the Institutional Review Board (IRB). The studies were conducted in accordance with the local legislation and institutional requirements. Written informed consent for participation in this study was provided by the participants’ legal guardians.

## Author contributions

SA: Conceptualization, Writing – original draft. ME: Conceptualization, Data curation, Writing – original draft. DY: Methodology, Validation, Writing – original draft. NE: Methodology, Writing – original draft. AM: Methodology, Writing – original draft. NY: Formal analysis, Writing – original draft. AmE: Formal analysis, Writing – original draft. HH: Supervision, Writing – review & editing. EK: Writing – review & editing. AlE: Supervision, Writing – review & editing.
