# Supplementary material for: Integration of measurable residual disease by WT1 gene expression and flow cytometry identifies pediatric patients with high risk of relapse in acute myeloid leukemia
Source: Front Oncol. 2024 Apr 24;14:1340909. doi: 10.3389/fonc.2024.1340909 (PMC11077298; doi:10.3389/fonc.2024.1340909)
Supplement: Supplementary file 1 [file DataSheet_1.docx]

**Legends for Supplementary Material**

**Supplementary Table 1:** Treatment Protocol Roadmap adopted from modified COG AAML 1031

**Supplementary Table 2:** Oligonucleotide primer and probe sequences for *WT1* and *ABL* genes used for quantitative real-time PCR

**Supplementary Table 3:** Impact of MRD response by *WT 1* after Induction I and Intensification I on survival among the whole cohort

**Supplementary Table 4:** Cox regression for univariate and multivariate analyses of MRD by *WT1* post intensification 1, FAB, *KMT2A*-r, favorable cytogenetic, unfavorable cytogenetic, and risk stratification post induction 1, A) Overall survival, B) event-free survival

**Supplementary Table 5:** Impact of MRD response by *WT1* on relapse among the 75 patients with MRD by MFC >0.1% post-induction I

**Supplementary Table S6:** Impact of MRD response by *WT1* on relapse among 66 patients with MRD by MFC <0.1% at the end of intensification I

**Supplementary Table 7:** Description of MRD response by MFC and *WT1* and PCR post Intensification I among low-risk with CBF and its impact on relapse

**Supplementary Table 1:** Treatment Protocol Roadmap adopted from modified COG AAML 1031


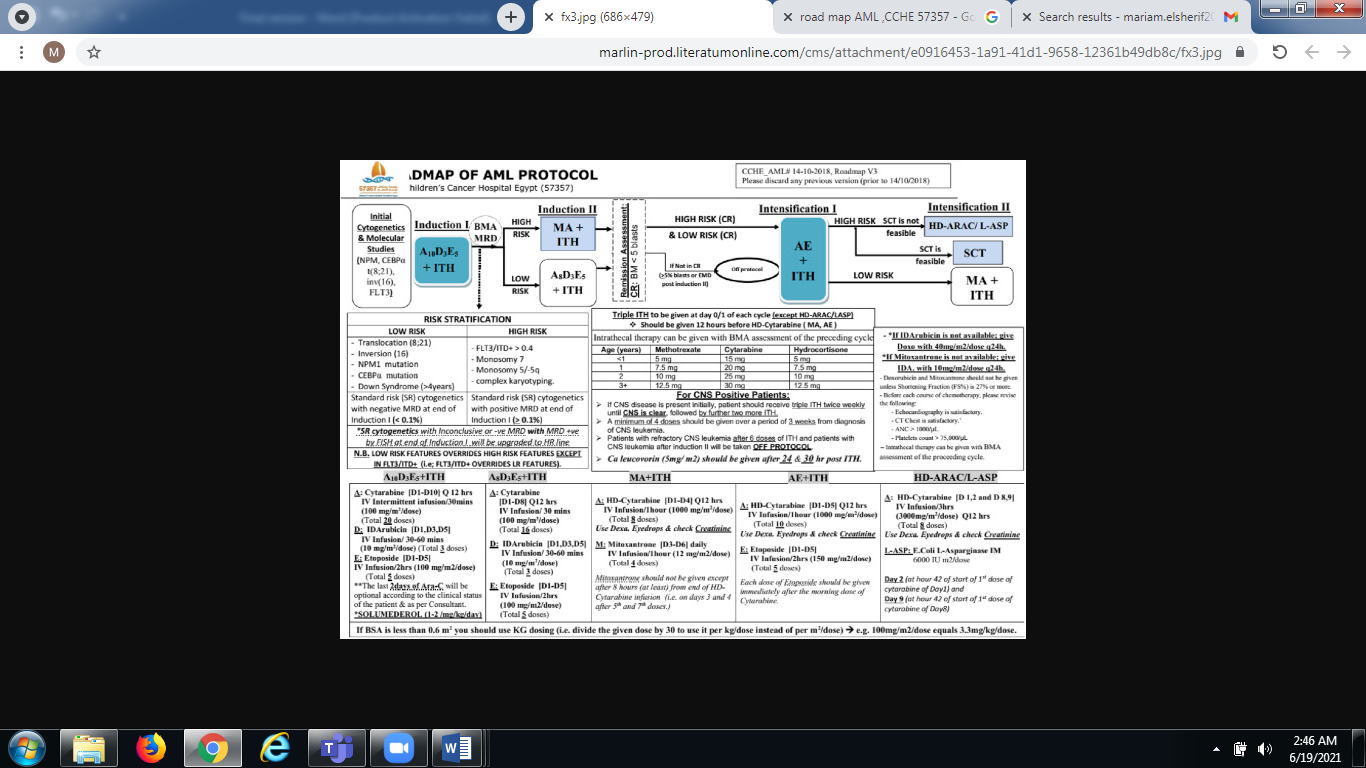


**Supplementary Table (2):** Primers and probe sequences for *WT1* and *ABL* genes

| **Primer/probes** | **Sequence (5'-3')** |
| --- | --- |
| *ABL*-F | 5'-TggAgATAACACTCTAAgCATAACTAAAggT-3' |
| *ABL*-R | 5'-gATgTAgTTgCTTgggACCCA-3' |
| *ABL*-probe | 5'-CCATTTTTggTTTgggCTTCACACCATT-3' |
| *WT1*-F | 5’CGC TAT TCGCAATCA GGG TTA 3’ |
| *WT1*-R | 5’ GGGCGTGTGACCGTAGCT3’ |
| *WT1*-probe | 5’AGCACGGTCACCTTCGACGGGA 3’ |

**Supplementary Table (3):** Impact of MRD response by *WT 1* after induction I and Intensification I on survival among the whole cohort

|  | **Total N** | **N of events** | **2-year OS** | **P-value** | **2-year EFS** | **N of events** | **P-value** | **2-year CIR** | **P-value** |
| --- | --- | --- | --- | --- | --- | --- | --- | --- | --- |
| **MRD by WT 1 post Ind1**  -Poor responder (<2 log)  -Good responder (≥2 log) | 49  38 | 15  7 | 68.9%  81.6% | 0.277 | 56.6%  73.7% | 22  11 | 0.178 | 33.2%  7.9% | **0.008^*^** |
| **MRD by WT 1 post Int 1**  -Poor responder (<2 log)  -Good responder (≥2 log) | 21  59 | 11  4 | 47.6%  93.2% | **<0.001^*^** | 33.3%  82.9% | 15  11 | **<0.001^*^** | 66.6%  10.6% | **<0.001^*^** |

*Significant; P-value < 0.05

- OS; overall survival, EFS; event free survival, CIR; cumulative incidence of relapse

- Ind I; induction I, Int 1; intensification I

**Supplementary Table 4:** Cox regression for univariate and multivariate analyses of MRD by *WT1* post intensification 1, FAB, *KMT2A*-r, favorable cytogenetic, unfavorable cytogenetic, and risk stratification post induction 1, A) OS, B) EFS

A)

| Overall Survival | Univariate | | | | Multivariate | | | |
| --- | --- | --- | --- | --- | --- | --- | --- | --- |
| Variable | **Hazards ratio** | **95%Confidence interval** | | **P-value** | **Hazards ratio** | **95% Confidence interval** | | **P-value** |
| MRD by *WT1* post int 1  (<2 vs >=2 ) | 6.738 | 2.062 | 22.01 | **0.002** | 3.002 | 0.896 | 10.064 | **0.04*** |
| Age | 0.996 | 0.915 | 1.021 | 0.22 |  |  |  |  |
| TLC group ( <50 vs >50 ) | 1.24 | 0.585 | 2.628 | 0.575 |  |  |  |  |
| M7 vs Non M7 | 2.719 | 1.395 | 5.302 | 0.003 |  |  |  |  |
| favorable | 0.229 | 0.115 | 0.456 | <0.001 |  |  |  |  |
| Unfavorable | 2.714 | 1.451 | 5.077 | 0.002 |  |  |  |  |
| *KMT2A-r* | 1.447 | 0.522 | 4.009 | 0.477 |  |  |  |  |
| Risk after induction 1  (HR vs LR) | 4.731 | 2.073 | 10.79 | **<0.001** | 18.056 | 2.224 | 146.60 | **0.007*** |

**B)**

| Event Free Survival | Univariate | | | | Multivariate | | | |
| --- | --- | --- | --- | --- | --- | --- | --- | --- |
| Variable | **Hazards  ratio** | **95%Confidence**  **interval** | | **P-value** | **Hazards  ratio** | **95% Confidence**  **interval** | | **P-value** |
| MRD by *WT1* post int1  (<2 vs >=2 ) | 4.491 | 2.051 | 9.83 | **<0.001** | 3.115 | 1.350 | 7.186 | **0.008*** |
| Age | 0.974 | 0.927 | 1.022 | 0.278 |  |  |  |  |
| TLC group (<50 vs >50 ) | 1.132 | 0.809 | 1.583 | 0.469 |  |  |  |  |
| M7 vs Non M7 | 1.533 | 1.12 | 2.1 | **0.008** |  |  |  |  |
| Favorable | 0.307 | 0.179 | 0.528 | **<0.001** |  |  |  |  |
| Unfavorable | 3.07 | 1.763 | 5.344 | **<0.001** |  |  |  |  |
| *KMT2A-r* | 0.671 | 0.27 | 1.666 | 0.39 |  |  |  |  |
| Risk after induction 1  (HR vs LR) | 3.081 | 1.686 | 5.632 | **<0.001** | 2.835 | 1.178 | 6.822 | **0.02*** |

*Significant; P-value < 0.05

**Supplementary Table 5:** Impact of MRD response by *WT1* on relapse among the 75 patients with MRD by MFC >0.1% post-induction I

| ***WT1* MRD** | **Total No.** | **Relapse** | | **P value** |
| --- | --- | --- | --- | --- |
|  |  | Yes | No |  |
| - Poor responder  (< 2 log reduction) | 40 | 12 | 28 | **0.021*** |
| - Good responder  (≥ 2log reduction) | 35 | 3 | 32 |  |
| **Total** | 75 | 15 | 60 |  |

*Significant; P-value < 0.05

**Supplementary Table 6:** Impact of MRD response by *WT1* on relapse among 66 patients with MRD by MFC <0.1% at end of intensification I

|  | **Total No.** | **Relapse** | | **P value** |
| --- | --- | --- | --- | --- |
| ***WT1* MRD** |  | **Yes** | **No** |  |
| - Poor responder   (< 2 log reduction) | 15 | 9 (60%) | 6 (40%) | **<0.001*** |
| - Good responder   (≥ 2log reduction) | 51 | 6 (11.8%) | 45 (88.2%) |  |
| **Total** | 66 | 15 | 51 |  |

*Significant; P-value < 0.05

**Supplementary Table 7:** Description of MRD response by MFC and *WT1* and PCR post Intensification I among low-risk with CBF and its impact on relapse

| Response | **Total No.** | **Relapse**  Yes |
| --- | --- | --- |
| **MRD by MFC**   - ≥ 0.1% (+ve) - < 0.1% (-ve) | 3  37 | 0  7 (19%) |
| ***WT1* MRD**   - Poor responder   (< 2 log reduction) (+ve)   - Good responder   (≥ 2log reduction) (-ve) | 6  34 | 4 (67%)  3 (9%) |
| **MRD by PCR**   - < 3 log reduction - ≥ 3 log reduction | 6  34 | 3 (50%)  4 (11.7%) |
